# Linoleic acid enhances angiogenesis through suppression of angiostatin induced by plasminogen activator inhibitor 1

**DOI:** 10.1038/bjc.2011.434

**Published:** 2011-10-20

**Authors:** N Nishioka, T Matsuoka, M Yashiro, K Hirakawa, K Olden, J D Roberts

**Affiliations:** 1 Laboratory of Molecular Carcinogenesis, National Institute of Environmental Health Sciences, NIH, Research Triangle Park, NC 27709, USA; 2 Department of Surgical Oncology, Osaka City University Graduate School of Medicine, Osaka 545-8585, Japan

**Keywords:** gastric carcinoma, linoleic acid, plasminogen activator inhibitor 1, angiostatin, invasion

## Abstract

**Background:**

The intake of dietary fatty acids is highly correlated with the risk of various cancers. Linoleic acid (LA) is the most abundant polyunsaturated fat in the western diet, but the mechanism(s) by fatty acids such as LA modulate cancer cells is unclear. In this study, we examined the role of LA in various steps in gastric cancer progression.

**Methods:**

The difference in gene expression between LA-treated and untreated OCUM-2MD3 gastric carcinoma cells was examined by mRNA differential display. The involvement of candidate genes was examined by oligo- and plasmid-mediated RNA interference. Biological functions of several of these genes were examined using *in vitro* assays for invasion, angiogenesis, apoptosis, cell viability, and matrix digestion. Angiogenesis *in vivo* was measured by CD-31 immunohistochemistry and microvessel density scoring.

**Results:**

LA enhanced the plasminogen activator inhibitor 1 (PAI-1) mRNA and protein expression, which are controlled by PAI-1 mRNA-binding protein. LA-stimulated invasion depended on PAI-1. LA also enhanced angiogenesis by suppression of angiostatin, also through PAI-1. LA did not alter cell growth in culture, but increased dietary LA-enhanced tumour growth in an animal model.

**Conclusion:**

Our findings suggest that dietary LA impacts multiple steps in cancer invasion and angiogenesis, and that reducing LA in the diet may help slow cancer progression.

In the United States, obesity is a contributing factor in the over 90 000 deaths from cancer per year (Flegal *et al*, 2009). Two-thirds of adults in the United States are overweight or obese (Flegal *et al*, 2009), and the fundamental causes of this ‘obesity epidemic’ are sedentary lifestyles and high-fat diets (WHO Report, 2000). Current evidence suggests that one of the best strategies for prevention of obesity is a diet low in fat (Astrup, 2005).

Among different dietary fatty acids, linoleic acid (LA), the most abundant polyunsatured fat in the western diet (Trumbo *et al*, 2002), has been reported to promote invasion and cell growth of the MDA-MB-435 breast cancer cell line (Rose *et al*, 1995) and increase colon tumour number in the Apc^min/+^ mice model (Whelan and McEntee, 2004). However, LA also has been reported to have different effects on different cancer cell lines. For example, it reduces growth of SK-HEP liver cancer cells and LNCap prostate cancer cells, but has no effect on growth of 639V bladder cancer cells and MDA-MB-231 breast cancer cells (Maggiora *et al*, 2004). These results would appear to be inconsistent, but may in fact make sense when the mechanism of action of LA acid is understood. We are interested in uncovering the mechanisms by which LA induces its effects in cancer cells, in hopes of using this information to intervene in cancer progression. We have chosen as our model the human scirrhous gastric cancer.

A consistent increase in the incidence of the diffuse types of gastric carcinoma (Lauren classification) has been seen during the last 50 years in the United States (Henson *et al*, 2004). Scirrhous gastric cancer is one of these, and is characterised by diffusely infiltrating carcinoma with frequent peritoneal dissemination. The prognosis of patients with scirrhous gastric cancer is poor compared with that of the other types of gastric cancer (Yashiro *et al*, 1996), for which the survival rate has been increasing due to improved diagnostic techniques and new operative procedures.

We have previously shown that LA and arachidonic acid, common omega-6 dietary fatty acids, promote invasion and peritoneal metastasis of human scirrhous gastric carcinoma cells in a mouse model (Matsuoka *et al*, 2010). Furthermore, conjugated LA has been reported to inhibit invasion and growth of gastric cancer cell line SGC-7901 (Chen *et al*, 2003), supporting the idea that fatty acid-regulated pathways may be useful in altering cancer progression. However, the mechanisms by which these eicosanoid precursors effect tumour progression and metastasis are not well defined. The goals of this study were to clarify the mechanisms *in vitro* and *in vivo* by which gastric cancer progression is enhanced by LA.

## Materials and methods

### Cell lines and cell culture

An extensively peritoneal-seeding cell line, OCUM-2MD3, was established from parental OCUM-2M, using orthotopic tissue implantation in nude mice. The cell line was maintained in DMEM (Invitrogen Corporation, Frederick, MD, USA) supplemented with 10% heat-inactivated bovine serum (Gemini Bio-Products, Woodland, CA, USA), 100 IU ml^−1^ of penicillin, and 0.5 mM sodium pyruvate, at 37 °C in a humidified atmosphere containing 5% carbon dioxide. Human umbilical vein endothelial cells (HUVECs) were maintained in HAM's F-12K medium supplemented with 15% heat-inactivated bovine serum, 100 IU ml^−1^ of penicillin, and 500 ng ml^−1^ epidermal growth factor.

### Differential display

OCUM-2MD3 cells were cultured 24 h with either LA (30 *μ*M) or vehicle (ethanol). Total RNA was extracted by using RNeasy Midi kit according to the manufacturer's instructions (Qiagen Inc., Valencia, CA, USA). Differential display was performed according to the manufacturer's instructions (Seegen Inc., Rockville, MD, USA). Briefly, total RNA (50 *μ*g) was incubated for 30 min at 37 °C with 10 U *μ*l^−1^ DNase for removing the genomic DNA. Total RNA (3 *μ*g) and 2 *μ*l anchor ACP-T (10 *μ*M) were incubated at 80 °C for 3 min. RT buffer (5 × ) of 4 *μ*l, 5 *μ*l 2 mM dNTP, 20 U RNase inhibitor, and 200 U M-MLV reverse transcriptase were added and incubated at 42 °C for 90 min. PCR reactions were performed with one of A1–A20 arbitrary ACP primers by the following regimen: denaturation at 94 °C for 40 s, annealing at 65 °C for 40 s, and extension at 72 °C for 40 s. PCR products were separated by electrophoresis and stained with ethidium bromide. The differentially expressed bands were extracted from 2% agarose gel by using the QIAquick Gel Extraction Kit (Qiagen). PCR products were cloned using TOPO TA Cloning (Invitrogen). Plasmid DNA was extracted by Qiagen plasmid Maxi Kits. DNA sequencing reactions were performed with BigDye Terminator Cycle Sequencing Kit (Applied Biosystems, Foster City, CA, USA). Sequences were determined following gel electrophoresis by the DNA sequencing facility at NIEHS. Homology searches of the differentially expressed bands were performed using NCBI BLAST.

### PAI–RBP knockdown

Oligonucleotides for *plasminogen activator inhibitor 1–RNA binding protein (PAI–RBP)* interference were pre-designed and synthesised by Ambion Inc. (Austin, TX, USA). Three targets (sense sequence: 5′-GGCAGCAGAGAACAAGAAAtt-3′, 5′-GGAAUAAGACGAGUUGGAAtt-3′, and 5′-GGCUAUUCAAAAUAAGGACtt-3′) were chosen and mixed for experiments. Oligonucleotides for non-targeted knockdown (siCONTROL non-targeting siRNA) were designed and synthesised by Dharmacon Inc. (Chicago, IL, USA). Cancer cells were cultured and kept subconfluent in six-well plates. Either 400 pmol *PAI–RBP* knockdown oligo or negative control knockdown oligo, and 10 *μ*l lipofectamine 2000 (Invitrogen) were mixed, incubated for 20 min in 1 ml Opti-MEM (Invitrogen), and added to each well. Cancer cells were grown in serum-free medium, and the medium was changed 12 h after adding siRNA. Total RNA was extracted 6 and 24 h after adding siRNA. Reverse transcription was performed as described. Protein was extracted 48 h after adding siRNA.

### Real-time RT–PCR

Real-time PCR was performed using an ABI PRISM 7900HT (Applied Biosystems). Real-time RT–PCR fluorescence detection was performed in 96-well plates, using the TaqMan Universal PCR Master Mix and Assay-on-Demand Assay Mix (PAI-1: HS00167155ml, *GAPDH*: HS99999905ml, and *PAI–RBP*: HS 00854675qH; Applied Biosystems). PCR reactions were performed by the following regimen: hold at 95 °C for 10 min, amplification; 40 cycles of 92 °C for 15 s and 60 °C for 1 min. Amplification products were checked using SDS2.1 software (Applied Biosystems). Samples were compared using the comparative Ct method. The Ct value was measured in triplicate relative to time-matched vehicle-treated controls in three independent samples, and calculated after adjusting for GAPDH using 2^−ΔΔCt^, where ΔΔCt=ΔCt control–ΔCt treatment, and ΔCt=target gene Ct−GAPDH Ct.

### Western blotting

Whole-cell lysates were resolved by 10% SDS–PAGE and transferred to nitrocellulose membrane in Tris-glycine buffer containing 10% methanol. The membrane was blocked with 5% milk in TBS-T at room temperature for 1 h. Antibodies to PAI-1 or GAPDH (Santa Cruz Biotechnology Inc., Santa Cruz, CA, USA) were incubated with the membrane at room temperature for 2 h or at 4 °C for overnight, and the membrane was incubated with horseradish peroxidase-conjugated secondary antibody for 1 h. Immunoreactivity was detected using chemiluminescent substrate (SuperSignal West Pico Chemiluminescent Substrate, Pierce Biotechnology Inc., Rockford, IL, USA). Quantification was performed using NIH Image software.

### Elisa assay

PAI-1 secretion *in vitro* was measured by a human PAI-1 activity assay (Molecular Innovations Inc., Southfield, MI, USA). Cancer cells were incubated with vehicle, or with 10, 30 or 60 *μ*M LA for 24 h. Medium was collected and centrifuged at 100 **g**. Purification and concentration were performed using CENTRIPREP (Millipore Corporation, Billerica, MA, USA) according to the manufacturer's instructions. Measurements were corrected for the absorbance in vehicle-treated samples. The PAI-1 concentration in mouse serum was measured using a murine PAI-1 Total Antigen Assay (Molecular Innovations). Blood was mixed at a ratio of 9 : 1 with 0.1 M trisodium citrate and centrifuged at 3000 **g** for 15 min. Plasma was stored at −20 °C.

### PAI-1 RNA interference by oligonucleotides

Two oligonucleotides (5′-AAUGACCGACAUGUUCAGACA-3′ and 5′-AAGAUCGAGGUGAACGAGAGU-3′) were designed using an algorithm from Dharmacon, and synthesised by Dharmacon. Three oligonucleotides (5′-AAGGAUGAGAUCAGCACCACA-3′, 5′-AAGGUAUGAUCAGCAACUUGC-3′, and 5′-AAGGAAGAGAAGACAUUUGCC-3′) were designed using an algorithm from Ambion Inc., and synthesised by Ambion. Oligonucleotides for non-targeted knockdown (Silencer Negative Control #1 siRNA) were designed and synthesised by Ambion. Cancer cells were cultured subconfluently in 24-well plates. Either 40 pmol *PAI-1* knockdown oligo or negative-control knockdown oligo, and 1 *μ*l lipofectamine 2000 (Invitrogen) were mixed, incubated for 20 min in 100 *μ*l Opti-MEM (Invitrogen), and added to each well. Cancer cells were grown in serum-free medium, and the medium was changed 12 h after adding siRNA. Protein was extracted 48 h after adding siRNA.

### Invasion assay

Transwell cell-culture chambers equipped with a microporous membrane filter (Millipore Corporation) were used for the invasion assay. Each chamber was placed into a 24-well plate in 1 ml of DMEM, and the microporous membranes were coated with Matrigel (100 *μ*g per 200 *μ*l). OCUM-2MD3 cells were resuspended to a final concentration of 1 × 10^6^ cells ml^−1^ in DMEM one day after transfection. Tumour cell suspensions (200 *μ*l) were separately added onto the upper surface of the Matrigel-coated membrane and incubated for 3 days at 37 °C. The filters were fixed with ethanol and stained with haematoxylin. The tumour cells on the upper surface of the filters were removed by wiping with cotton swabs. The cells that had invaded through the Matrigel and filter to the lower surface were counted under a microscope. For each group, the assay was performed in triplicate, and numbers are reported as cells per microscopic field.

### PAI-1 RNA interference by transient transfection of plasmid vector

Hairpin siRNA template oligonucleotides for *PAI-1* were designed using an algorithm from Ambion and synthesised by Invitrogen. The one loop sequence is 5′-GATCCCGTGACCGACATGTTCAGACATTCAAGAGATGTCTGAACATGTCGGTCATTTTTTGGAAA-3′. The other sequence is 5′-AGCTTTTCCAAAAAATGACCGACATGTTCAGACATCTATTGAATGTCTGAACATGTCGGTCACGG-3′. The annealed hairpin siRNA oligonucleotides were inserted into p*Silencer* 4.1-CMV hygro vector (Ambion). The negative control was an siRNA template sequence that lacks significant homology to the mouse, human, and rat genome databases. The vectors were cloned into DH5α competent cells. OCUM-2MD3 cells were seeded in 12-, 24-, or 96-well plates and 100-mm dishes at 1 × 10^5^, 6 × 10^4^, 1 × 10^4^ cells per well and 6 × 10^6^ cells per dish, respectively, and grown overnight to ∼60% confluence before transfection. Cells were transfected with siPORT *XP-1* transfection reagent (Ambion). Appropriate amounts (400 ng (12-well), 200 ng (24-well), 40 ng (96-well), and 10 *μ*g (100 mm dish)) of the siRNA expression plasmids were used. One day after transfection, 200 *μ*g ml^−1^ of hygromycin B was added. All transfection experiments were done in triplicate.

### Immunofluorescence microscopy

Immunofluorescence localisation was performed in fixed and permeabilised cells adhered to 18-mm diameter glass cover slips in 12-well plates. Three days after transfection, cells were fixed in 3% paraformaldehyde and permeabilised for 5 min in PBS containing 0.1% triton X-100. Fixed cells were incubated for 1 h with mouse anti-PAI-1 antibody (Santa Cruz Biotechnology). Cells were incubated for 1 h with the secondary antibody, Alexa Fluor 594-conjugated donkey anti-mouse IgG (Invitrogen), and for 5 min with DAPI (Invitrogen). Cover slips were mounted on glass slides, adding ProLong Antifade (Invitrogen) to inhibit photobleaching. Immunofluorescence was documented with a Zeiss Axiovert 35 microscope.

### Apoptosis assay

The protocol was adapted from a DNA fragmentation detection kit (Calbiochem, San Diego, CA, USA). Briefly, 2 days after transfection, 1 × 10^6^ cells were washed with PBS, fixed with 4% formaldehyde for 10 min, and resuspended in 80% ethanol. Cells were labelled at 37 °C in the dark for 1 h with terminal TdT incubation buffer containing TdT enzyme and fluorescein-dNTP. The cells were analysed with a FACSort flow cytometer, measuring fluorescence at 488 nm.

### Angiogenesis assay

In this *in vitro* model of angiogenesis, HUVECs were grown in a co-culture with siRNA expression vector-transfected OCUM-2MD3 cells. Three days before the HUVECs culture, OCUM-2MD3 cells were seeded in the inner wells of a 24-well transwell dish (Costar, Cambridge, MA, USA); 24 h after seeding, siRNA expression plasmids were applied. When appropriate, ECMatrix (Chemicon International Inc., Temecula, CA, USA) was added (83 *μ*l) to each outer well of a 24-well transwell dish, and allowed to polymerise. A suspension of 2 × 10^4^ HUVECs in culture medium was added to the outer wells and incubated with the inner wells at 37 °C overnight. For blocking angiostatin receptors, HUVECs were preincubated 30 min with 100 *μ*g ml^−1^ of *α-* and *β*-subunits of ATP synthase antibodies (Invitrogen) or control mouse IgG (Invitrogen). When appropriate, Matrigel (BD Biosciences, San Jose, CA, USA) was added (100 *μ*l) to each outer well. OCUM-2MD3 cells were preincubated with PAI-1 antibody (American Diagnostica Inc., Stamford, CT, USA) or control mouse IgG (Invitrogen) for blocking PAI-1.

### Digestion assay of plasminogen to angiostatin by cell-culture supernatant

OCUM2-MD3 cells were cultured with SFCM. Purified human plasminogen (Sigma-Aldrich Corp., St Louis, MO, USA) was added to SFCM at a final concentration of 25 *μ*g ml^−1^ and incubated at 37 °C for 7 h with PAI-1 antibody (0, 5, 10, 15, or 30 *μ*g ml^−1^) or control mouse IgG_1_. SFCM/plasminogen of 15 *μ*l was analysed by gel electrophoresis. Western blots were performed as described above. Antibodies to angiostatin (EMD Biosciences Inc., San Diego, CA, USA) were incubated with the membrane at 4 °C overnight.

### *In vivo* model for gastric carcinoma

Four-week-old female athymic nude mice were obtained from Charles River Laboratories (Raleigh, NC, USA) and maintained in microisolator cages in a specific pathogen-free facility. All studies were approved by the NIEHS Animal Care and Use Committee, and conducted in accordance with PHS guidelines and the National Research Council Guide for the Care and Use of Laboratory Animals. Diets were based on the purified AIN-76A diet and prepared by BioServe Inc. (Frenchtown, NJ, USA). The two diets contained the same high-fat content (23%, w/w), but varied in content of LA-rich safflower oil and saturated FA-containing coconut oil in ratios to yield either 2 or 12% (w/w) LA. Feeding of the experimental diets commenced 7 days before injecting the tumour cells. Mice were divided into four groups (10 animals per group), which were fed 2 or 12% LA-containing diet, then injected subcutaneously or intraperitoneally with OCUM-2MD3 cells (2 × 10^6^ cells in 200 *μ*l serum-free DMEM). Mice were killed by CO_2_ euthanasia 4 weeks after cancer cell injection. At necropsy, whole blood was collected and the extent of tumour growth and/or metastasis was assessed. Tumour volumes were calculated using the equation *a* × *b*^2^ × 0.5, in which *a* and *b* are the largest and smallest diameters, respectively. Mouse serum angiostatin was detected by rabbit anti-mouse angiostatin antibody (Yanaihara Institute Inc., Shizuoka, Japan).

### Immunohistochemistry and microvessel density scoring for angiogenesis *in vivo*

The streptavidin–biotin peroxidase complex method was used for immunohistochemistry. Slides of tissue samples were treated with 0.3% hydrogen peroxide in methanol for 20 min. Sections were incubated for 2 × 30 min with anti-CD31 antibodies (Santa Cruz Biotechnology) diluted 1/750 in Dako Antibody Diluent (Dako Cytomation, Carpinteria, CA, USA). The slides were counterstained with Mayer's haematoxylin for 1 min and washed with water. Histological slides were blind-coded during assessment. The tissue sections were viewed at × 100 magnification (0.565 mm^2^ per field). Four fields per section were randomly analysed. MVD (i.e., the number of CD31-positive objects/0.565 mm^2^) was calculated.

### Statistical analysis

The data were analysed using Student's *t*-test and Mann–Whitney's *U*-test. A *P*-value of less than 0.05 was considered statistically significant.

## Results

### Differential display suggests PAI-1 mRNA-binding protein is a candidate gene induced by LA

**Figure 1:**
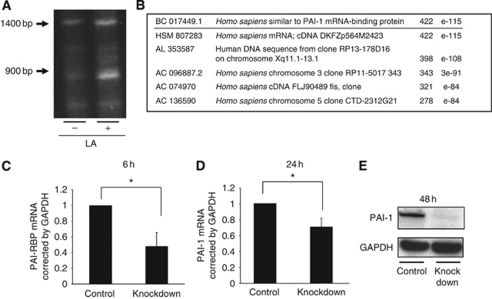
Differential display of OCUM-2MD3 cells treated with LA and vehicle. (**A**) Differential display of the amplified cDNA products from LA-treated cells and vehicle-treated cells. Arrows indicate base pair. (**B**) Homology results of the differential display band by NCBI BLAST. PAI-1 mRNA-binding protein shows highest score. (**C**) PAI–RBP mRNA expression 6 h after oligo RNAi and control RNAi treatment as measured by real-time RT–PCR. PAI–RBP mRNA expression was suppressed to 48% by oligo RNAi. (**D**) PAI-1 mRNA expression 24 h after PAI–RBP, and control oligo RNAi treatment as measured by real-time RT–PCR. PAI-1 mRNA expression was suppressed to 70% by PAI–RBP knockdown. Asterisk indicates *P*<0.05. Values shown are mean±s.d. (**E**) PAI-1 protein expression 48 h after PAI–RBP and control oligo RNAi treatment. PAI-1 protein expression was suppressed by PAI-1 mRNA-binding protein knockdown.

We used two methods, differential display and microarray (data not shown), for identifying potential genes that may be induced by LA to have a role in cancer progression. A differentially expressed band appeared around 900 bp in the PCR products with primer number 12 (Figure 1A). *PAI–RBP* showed the highest score in a homology search by BLAST (Figure 1B). Given the recent finding that patients with low expression of uPA/PAI-1 had a significantly lower risk of disease recurrence (Mulligan-Kehoe *et al*, 2006), we hypothesised that control of PAI-1 is important for cancer progression enhanced by LA, and chose *PAI–RBP* as a candidate gene induced by LA for further study.

### RNA interference suppresses PAI-1 mRNA and protein expression

**Figure 2:**
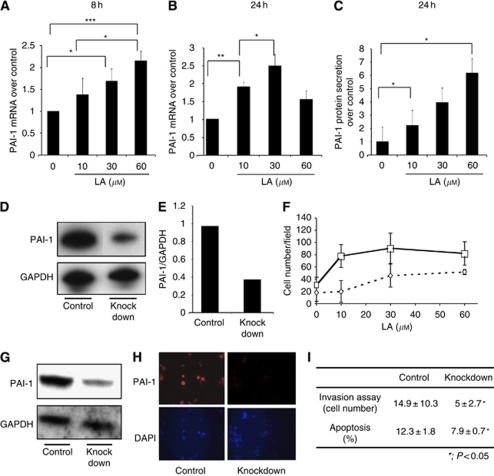
PAI-1 expression treated with LA. (**A**) PAI-1 mRNA expression 8 h after treatment with LA. Changes in gene expression were measured by real-time RT–PCR, and are expressed over control corrected for GAPDH mRNA levels. (**B**) PAI-1 mRNA expression 24 h after treatment with LA. Asterisk indicates *P*<0.05. A double asterisk indicates *P*<0.01. A triple asterisk indicates *P*<0.001. Values shown are mean±s.d. (**C**) PAI-1 protein secretion in medium 24 h after treatment with LA. PAI-1 protein was measured by ELISA assay. Asterisk indicates *P*<0.05. Values shown are mean±s.e. (**D**) Western blotting of PAI-1 knockdown and control knockdown OCUM-2MD3 cells. PAI-1 protein expression was decreased by PAI-1RNAi oligo. The top panel shows western blotting of PAI-1. The bottom panel shows western blotting of GAPDH. (**E**) Quantification of PAI-1 knockdown efficiency by using NIH image software. PAI-1 protein expression was reduced to 40%. (**F**) The effect of PAI-1 knockdown by oligo RNAi on invasion ability. Solid line with squares: treated with LA and control oligo RNAi; broken line with diamonds: treated with LA and PAI-1 oligo RNAi. Statistical difference between control and PAI-1 oligo RNAi-treated responses was statistically significant (*P*<0.0001 by the Cochran–Mantel–Haenszel test). Values shown are mean±s.d. (**G**) PAI-1 protein knockdown by siRNA expression vector. The top panel shows western blotting of PAI-1. The bottom panel shows western blotting of GAPDH. (**H**) Immunofluorescence microscopy images ( × 200 magnification) of PAI-1 knockdown cells and control knockdown cells. Top panel shows PAI-1 staining. Bottom panel shows DAPI staining. (**I**) The effect of PAI-1 siRNA expression vector on invasion (by transwell assays) and apoptosis. Asterisk indicates *P*<0.05. Values shown are mean±s.d.

PAI–RBP is reported to regulate mRNA stability of PAI-1 (Heaton *et al*, 2003); however, there is no data on whether this protein increases or decreases PAI-1 expression in gastric carcinoma. Therefore, we tried to clarify the functional relationship between these proteins by using RNA interference. Knockdown efficiency of *PAI–RBP* was confirmed by real-time RT–PCR and showed that mRNA levels decreased to 48% (Figure 1C). Knockdown of *PAI-1* mRNA-binding protein also led to a decrease of both *PAI-1* mRNA (Figure 1D) and protein (Figure 1E). Thus, *PAI-1* mRNA, PAI-1 protein, and PAI–RBP mRNA were all reduced by *PAI–RBP* knockdown. These data suggested that LA increased mRNA expression of the protein, which stabilised *PAI-1* mRNA, so we examined whether LA led to an increase in PAI-1 expression. LA significantly enhanced *PAI-1* mRNA expression at both 8 h (Figure 2A) and 24 h (Figure 2B), and PAI-1 protein secretion at 24 h (Figure 2C).

### Dietary LA enhances PAI-1 concentration in serum and tumour growth *in vivo*

We have previously described *in vivo* metastasis studies in which mice fed diets containing LA at 12% showed increased tumour incidence, frequency, and volume of tumour nodules per mouse, when compared with mice fed diets containing LA at 2% (Matsuoka *et al*, 2010). LA enhanced PAI-1 expression *in vitro*; thus, we examined whether LA in the diet enhanced PAI-1 expression and correlated with tumour growth *in vivo*. We chose a subcutaneous injection model for tumour growth measurement. Five weeks after starting the mice on a diet of either 2 or 12% LA, PAI-1 serum concentration and subcutaneous tumour size were measured. LA significantly enhanced PAI-1 serum concentration (3.21±0.79 *vs* 7.60±1.17 ng ml^−1^, *P*<0.01) and tumour size (344.1±115 *vs* 1172.5±266.5 mm^3^, *P*<0.01). Thus, dietary LA appears to significantly enhance PAI-1 expression in serum and gastric carcinoma tumour growth *in vivo*.

### PAI-1 RNA interference blocks invasion ability enhanced by LA

Next, we examined whether PAI-1 has a role in key steps in cancer metastasis using an *in vitro* invasion model and oligo-mediated RNA interference. We chose the two most effective targets (5′-AAUGACCGACAUGUUCAGACA-3′ and 5′-AAGAUCGAGGUGAACGAGAGU-3′) for the knockdown of *PAI-1* among five targets tested (data not shown). Knockdown efficiency was confirmed by western blotting (Figure 2D) and quantification (Figure 2E). PAI-1 protein expression was decreased to 37% of control values.

We previously showed that *in vitro*, LA stimulated invasion of OCUM-2MD3 cells through extracellular matrix, and that this process may be related to the ERK pathway and cyclooxygenase activity (Matsuoka *et al*, 2010). In the current studies, *PAI-1* RNA interference significantly inhibited the invasion ability of OCUM-2MD3 (*P*<0.001; Figure 2F). Similar results were obtained using antibody to PAI-1 (data not shown). Thus, the LA-enhanced invasion was sensitive to inhibition of *PAI-1*, suggesting that PAI-1 has a critical role in the invasion induced by LA *in vitro*.

**Figure 3:**
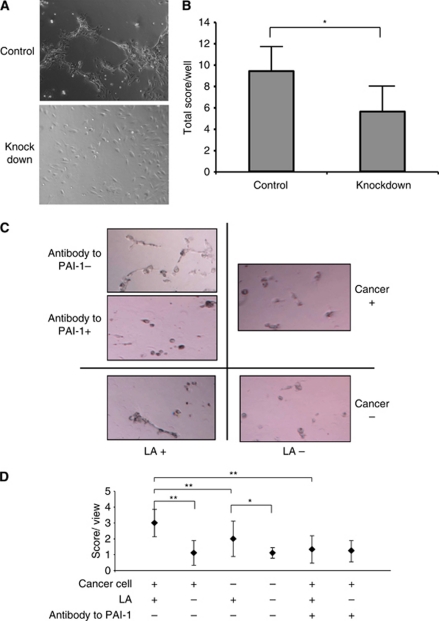
Angiogenesis assay of HUVECs in co-culture with control or PAI-1-knockdown siRNA expression vector-transfected OCUM-2MD3 cells. (**A**) Representative images of each group ( × 100 magnification). (**B**) Quantification of angiogenesis assay. Five random viewfields per well in five wells were examined in triplicate. Asterisk indicates *P*<0.01. Values shown are mean±s.d. (**C**) Representative images of angiogensis assay of HUVECs in co-culture with and without cancer cells treated with 30 *μ*M LA or vehicle. Cancer cells were preincubated with 30 *μ*g ml^−1^ antibody to PAI-1 or vehicle. HUVECs and cancer cells were treated with LA or vehicle for 24 h. (**D**) Quantification of angiogenesis assay. LA-enhanced HUVECs’ angiogenesis. Cancer cells have synergistic effects on LA-enhanced HUVECs’ angiogenesis (*P*<0.01). Antibody to PAI-1 inhibited LA-enhanced angiogenesis (*P*<0.01). Four random viewfields per well in three wells were examined. The data represent one experiment from two independent experiments. Values shown are mean±s.d. Asterisk indicates *P*<0.05. A double asterisk indicates *P*<0.01.

Plasmid vector-mediated *PAI-1* RNA interference inhibits HUVECs’ angiogenesis. We next examined the effect of plasmid vector-mediated RNA interference on PAI-1 protein expression in OCUM-2MD3 cells for *in vivo* use. Five siRNA target sites were tested within the coding region of the *PAI-1* mRNA. We chose one target, which most effectively knocked down the protein expression as viewed by western blotting analysis and immunofluorescence microscopy images (Figure 2G and H). Plasmid vector-mediated *PAI-1* RNA interference significantly decreased invasion ability and apoptosis (*P*<0.05 and *P*<0.05; Figure 2I). However, there was no difference in cell viability between the two groups (data not shown). One possible explanation for changes in tumour growth *in vivo*, without changes in cell growth *in vitro*, is alterations in the microenvironment within the animal. Thus, we hypothesised that a critical process providing nutrients to the tumour, angiogenesis, might be involved. We therefore examined the effect of *PAI-1* knockdown on angiogenesis. We chose a model of co-culture of HUVECs with OCUM-2MD3 cells. In this assay, we observed that angiogenic structures were decreased in a co-culture of HUVECs with *PAI-1*-knockdown OCUM-2MD3 cells, compared with the same co-culture with control-knockdown OCUM-2MD3 cells (*P*<0.05, Figure 3B). Typical images in each group were shown in Figure 3A.

The *in vitro* roles of PAI-1 in cancer progression were examined and next, we tried to clarify the relationship between LA and PAI-1 effect on angiogenesis *in vitro*. Typical images are shown in Figure 3C LA enhanced HUVECs’ angiogenesis even in the absence of cancer cells (*P*<0.05; Figure 3D). Co-culture with cancer cells had a synergistic effect on angiogenesis (*P*<0.01; Figure 3D). Furthermore, antibody to PAI-1 reduced angiogenesis enhanced by LA and cancer cells (*P*<0.01, Figure 3D). Thus, LA appears to enhance angiogenesis through a mechanism that depends on PAI-1.

### Blocking PAI-1 enhances angiostatin secretion in medium, and blocking angiostatin receptor restores angiogenesis suppressed by blocking PAI-1

**Figure 4:**
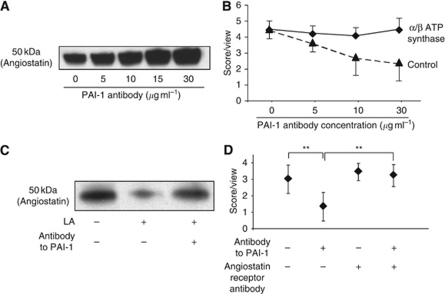
Angiostatin expression and angiogenesis assay with changing concentration of antibody to PAI-1. (**A**) Digestion assay of plasminogen to angiostatin by cell-culture supernatant. (**B**) Angiogenesis assay of HUVECs with blocking angiostatin receptors and control. Four random viewfields per well in three wells were examined. HUVECs were preincubated 30 min with 100 *μ*g ml^−1^ *α-*/*β*-subunits of ATP synthase antibodies (♦) or control mouse IgG (▴) before plating the cells. The data represent one experiment from two similar experiments. Values shown are mean±s.e. (**C**) LA decreased angiostatin expression. Antibody to PAI-1 restored reduction of angiostatin expression in medium by LA. (**D**) Angiostatin-receptor blocking recovered reduction of HUVECs’ angiogenesis by PAI-1 antibody (*P*<0.01). Four random viewfields per well in three wells were examined. The data represent one experiment from two independent experiments. Values shown are mean±s.d. A double asterisk indicates *P*<0.01.

We next tried to clarify the mechanism by which LA enhanced angiogenesis through PAI-1. First, we checked VEGF secretion from OCUM-2MD3 cells, but no difference was detected between PAI-1-blocking and control groups (data not shown). Second, angiostatin expression was examined. Angiostatin is reported to be altered by a truncated PAI-1 protein (Mulligan-Kehoe *et al*, 2002). The inhibition of PAI-1 enhanced angiostatin secretion in a dose-dependent fashion (Figure 4A). Blocking the angiostatin receptor restored the angiogenesis stimulating effect that was suppressed by PAI-1 antibody (Figure 4B). Thus, it appears that PAI-1 controls angiogenesis through angiostatin.

### Angiostatin expression is suppressed by LA, and blocking the angiostatin receptor restores the LA-induced angiogenesis suppressed by PAI-1 antibody

Next, we investigated the LA effect on angiostain expression and the relationship between PAI-1 and angiostatin in angiogenesis enhanced by LA. LA decreased angiostatin expression and antibody against PAI-1 restored angiostatin expression (Figure 4C). We examined HUVECs’ angiogenesis in a co-culture with cancer cells to which 30-*μ*M LA was added. Antibody to PAI-1 suppressed angiogenesis induced by LA (*P*<0.01; Figure 4D), and blocking the angiostatin receptor restored the anti-angiogenesis effect of blocking PAI-1 (*P*<0.01; Figure 4D). However, PAI-1 had no effect on angiogenesis when angiostatin receptor was blocked (Figure 4D). Together, these data indicate that LA enhances angiogenesis through suppression of angiostatin, and that PAI-1 has a critical role, involving the regulation of angiostatin in this fatty acid-induced process.

### Dietary LA suppresses serum angiostatin expression and enhances angiogenesis *in vivo*

**Figure 5:**
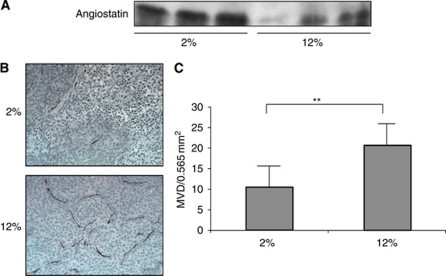
Angiostatin expression in serum and microvessel density in tumours from mice fed either 2 or 12% LA diets. (**A**) Angiostatin expression in 2 and 12% LA-fed mice. (**B**) Representative images of MVD in tumours of 2 and 12% LA-fed mice. Brown colour indicated CD 31-positive objects. (**C**). Quantification of MVD in tumours of 2 and 12% LA-fed mice. Values shown are mean±s.d. A double asterisk indicates *P*<0.01. The colour reproduction of this figure is available at the *British Journal of Cancer* online.

We previously reported that LA enhanced tumour incidence, volume, and number in a peritoneal metastasis model of gastric carcinoma. In this study, we extended our work-examined LA effects on angiostatin expression and angiogenesis in this *in vivo* mouse model. Mice were fed either 2 or 12% LA-containing diets for 5 weeks, at which time blood was collected and serum assayed for antiostatin. Tumour burden was examined 4 weeks after peritoneal injection of gastric carcinoma cells. Angiostatin expression in serum from mice with fed 12% LA was lower than that in serum from mice fed with 2% LA-containing diet (Figure 5A). CD-31 staining of tumour tissue revealed that MVD in 12% LA-fed mice (20.64±9.32/0.565 mm^2^) was significantly greater than that in 2% LA-fed mice (10.45±8.6/0.565 mm^2^; *P*<0.01; Figure 5C). Typical images of CD31 staining in each group are shown in Figure 5B. These data suggest that angiostatin expression was suppressed by dietary LA, thus leading to enhanced-tumour angiogenesis *in vivo*.

## Discussion

We have shown that the ability to stimulate angiogenesis is significantly increased when human gastric carcinoma cells are treated with LA without affecting cell growth *in vitro*, and that, angiostatin controlled by PAI-1 has an important role in angiogenesis enhanced by LA. To our knowledge, this is the first report that identifies such a significant correlation, and defines some of the key mechanisms, between LA and angiogenesis in a gastric carcinoma model.

LA has been reported to enhance the secretion of PAI-1 by HepG2 cells (Banfi *et al*, 1997) and 15d-PGJ_2_, downstream of LA, and to increase PAI-1 expression in endothelial cells through PPARγ activation (Marx *et al*, 1999). Furthermore, PAI-1 has been found to be elevated in obese humans (Juhan-Vague and Alessi, 1997). This combination of our results and these reports suggest that changes in dietary habits could be used to reduce cancer progression.

PAI–RBP has been reported to regulate *PAI-1* mRNA stability (Heaton *et al*, 2003), but there is no data on whether this binding protein increases or decreases *PAI-1* mRNA and protein. In this study, we found that LA enhanced PAI-1 secretion in part, at least, controlled by PAI–RBP at the post-transcriptional level. However, the mechanism by which PAI–RBP functions to increase PAI-1 is still unclear. Further investigations of interactions between PAI–RBP and PAI-1 are needed to identify their precise roles in the development and progression of gastric cancer.

Host-derived PAI-1 is critical for *in vivo* angiogenesis (Bajou *et al*, 1998) although PAI-1 produced by tumour cells did not overcome the absence of PAI-1 in the host (Bajou *et al*, 2004). Nevertheless, our assays for angiogenesis showed that *PAI-1* knockdown in cancer cells reduced angiogenesis *in vitro*, suggesting that tumour cell-derived PAI-1 is also critical for tumour angiogenesis. A truncated PAI-1 has been reported to induce and inhibit angiostatin (Mulligan-Kehoe *et al*, 2001). Three angiostatin receptors has been reported: *α-*/*β*-ATP synthase (Moser *et al*, 1999), angiomotin (Troyanovsky *et al*, 2001), and integrin*α*_V_*β*_3_ (Tarui *et al*, 2001). ATP synthase antibodies and integrin*α*_V_*β*_3_ successfully blocked angiostatin receptors in our studies, but blocking integrin*α*_V_*β*_3_ actually reduced angiogenesis (data not shown). This may be due to the blocking effect of integrin*α*_V_*β*_3_ on HUVECs’ migration (Tarui *et al*, 2002), a step that is critical for angiogenesis. Interestingly, angiogenesis in a co-culture model of HUVECs with plasmid vector-transfected OCUM-2MD3 cells seems to be lower than that in experiments in which the antibody was added. At this time, we cannot rule out cytotoxic effects of the tranfection reagent, but are continuing to attempt to define the mechanisms by which angiogenesis is stimulated by LA in this model. It does appear, however, that angiostatin has an important role in the angiogenesis induced by LA *in vitro* and *in vivo*.

Our results with the invasion assay indicated that PAI-1 inhibition suppressed invasion of OCUM-2MD3 cells. In contrast, Praus *et al* (1999) showed a reduction of HT 1080 fibrosarcoma cell invasion through matrigel by adenoviral transfer of PAI-1, and Whitley *et al* (2004) showed a reduction of MDA-MB-435 cell invasion after overexpression of PAI-1. However, XR5967, an inhibitor of PAI-1, is reported to suppress HT1050 cell invasion (Brooks *et al*, 2004), and an absence of host PAI-1 prevents PVDA keratinocyte invasion (Bajou *et al*, 2004). These results indicate, depending on the cell type and the precise condition, PAI-1 shows different effects on invasion ability of cancer cells. In general, PAI-1 concentrations near the normal physiological range appear to promote invasion, whereas high concentrations of exogenous PAI-1 appear to inhibit invasion.

Our data suggest that *PAI-1* RNAi has little effect on apoptosis and viability of OCUM-2MD3. This result is different from that of Kwaan *et al* (2000) who showed PAI-1 promoted tumour growth through inhibition of apoptosis in the prostate cancer cell line PC-3 and leukaemia cell HL-60. In addition, Chen *et al* (2004) showed inhibition of apoptosis in vascular smooth muscle cells by PAI-1. Conversely, Isogai *et al* (2001) reported that PAI-1 did not induce endothelial cell apoptosis. TGF-*β* is known to be upstream of PAI-1 (Song *et al*, 1998) and to enhance growth of OCUM-2MD3 cell (Yashiro *et al*, 1995), and inhibit or enhance apoptosis, depending on the cell types (Siegel and Massague, 2003). We found that siRNA inhibition of *PAI-1* in OCUM-2MD3 cells enhanced TGF-*β* protein expression (data not shown). Thus, there may be negative feedback loops between PAI-1 and TGF-*β*. PAI-1 is reported to inhibit apoptosis through caspase-3 (Chen *et al*, 2004) and TGF-*β* is thought to induce apoptosis through SMAD, JNK, and caspase-3 (Siegel and Massague, 2003). This combination of signalling and feedback may explain the difference in results from different labs using different cell types.

Taken together, our data indicate that LA enhances *PAI-1* mRNA and protein expression in gastric carcinoma cells, in a manner controlled by PAI-1 mRNA-binding protein. LA increases invasion and angiogenesis by suppression of angiostatin through PAI-1 expression, though the mechanism of this process is not yet clear. The effects on angiostatin may also explain the increased growth of tumour xenografts *in vivo*, whereas having no effect on growth properties of the cells in culture. These findings suggest that LA in the diet contributes to cancer invasion and angiogenesis through multiple pathways. Thus, reducing the LA in the diet may be an effective way to slow cancer progression by suppression of PAI-1 and enhancement of angiostatin.
